# Hitting the Bull’s-Eye in Metastatic Cancers—NSAIDs Elevate ROS in Mitochondria, Inducing Malignant Cell Death

**DOI:** 10.3390/ph8010062

**Published:** 2015-02-13

**Authors:** Stephen John Ralph, Rhys Pritchard, Sara Rodríguez-Enríquez, Rafael Moreno-Sánchez, Raymond Keith Ralph

**Affiliations:** 1 School of Medical Science, Griffith University, Griffith Health Institute, Parklands Drive, Southport, Gold Coast, Queensland 4222, Australia; 2 Instituto Nacional de Cardiología, Departamento de Bioquímica, Tlalpan, México D.F., Mexico; 3 Instituto Nacional de Cancerología, Laboratorio de Medicina Translacional, Tlalpan, México D.F., Mexico

**Keywords:** cancer cells, metastasis, mitochondrial permeability transition pore, non-steroidal anti-inflammatory drugs, reactive oxygen species

## Abstract

Tumor metastases that impede the function of vital organs are a major cause of cancer related mortality. Mitochondrial oxidative stress induced by hypoxia, low nutrient levels, or other stresses, such as genotoxic events, act as key drivers of the malignant changes in primary tumors to enhance their progression to metastasis. Emerging evidence now indicates that mitochondrial modifications and mutations resulting from oxidative stress, and leading to OxPhos stimulation and/or enhanced reactive oxygen species (ROS) production, are essential for promoting and sustaining the highly metastatic phenotype. Moreover, the modified mitochondria in emerging or existing metastatic cancer cells, by their irreversible differences, provide opportunities for selectively targeting their mitochondrial functions with a one-two punch. The first blow would block their anti-oxidative defense, followed by the knockout blow—promoting production of excess ROS, capitulating the terminal stage—activation of the mitochondrial permeability transition pore (mPTP), specifically killing metastatic cancer cells or their precursors. This review links a wide area of research relevant to cellular mechanisms that affect mitochondria activity as a major source of ROS production driving the pro-oxidative state in metastatic cancer cells. Each of the important aspects affecting mitochondrial function are discussed including: hypoxia, HIFs and PGC1 induced metabolic changes, increased ROS production to induce a more pro-oxidative state with reduced antioxidant defenses. It then focuses on how the mitochondria, as a major source of ROS in metastatic cancer cells driving the pro-oxidative state of malignancy enables targeting drugs affecting many of these altered processes and why the NSAIDs are an excellent example of mitochondria-targeted agents that provide a one-two knockout activating the mPTP and their efficacy as selective anticancer metastasis drugs.

## 1. Introduction

Although improvements in treating advanced stages of highly malignant cancers are now being made using monoclonal antibodies and personalized medicine with kinase inhibitors [1,2], mortality from metastatic disease continues to rise and remains a major health issue [3]. Hanahan and Weinberg redefined and updated the hallmarks of cancer [4] to promote the concept of epithelial-mesenchymal transition (EMT) as an essential step prior to primary tumor invasion and metastasis. However, they omitted recognizing the importance of hypoxia (and hypoglycemia) and their effects on mitochondrial function as the main underlying drivers for the process of metastatic tumor progression [5,6].

Tumor hypoxia is a major problem for radiation therapy, but it is also involved in resistance to many chemotherapies because it greatly alters gene expression, an important driver of tumor development and the progression to metastasis [6,7]. Hypoxic microenvironments frequently exist in solid tumors [8] with O_2_ levels fluctuating temporally and spatially from normoxic (10%–21% atmospheric O_2_) to hypoxic (0.1%–5% O_2_) [9]. This is due to the instability and chaotic nature of the tumor vasculature as solid tumors continually undergo hypoxic stress caused when rapidly dividing cancer cells outgrow their vascular networks, diminishing the local supply of O_2_. Hypoxic stress also results from abnormal tumor blood vessel structure and function [10] and it induces tissue necrosis and cell selection, with many cells subsequently dying, while also significantly altering tumor cell biology, decreasing sensitivity to apoptotic or other cell-death signals in surviving cells, and increasing signals promoting angiogenesis, proliferation and metastasis [11].

It should be noted that the great majority of studies on hypoxia and cancer cells have been performed in the presence of high glucose. However, hypoxic events in solid tumors are accompanied by hypoglycemia, decreasing the external glucose concentration from around 5 mM down to about 0.01–2.8 mM [12]. It is expected that hypoglycemia may potentiate the hypoxia effects, but studies using both stresses have not been undertaken.

Hypoxia increases tumor-initiating or cancer stem cell (CSC) numbers in many cancers as small subsets of stem-like cells showing a greater capacity for self-renewal, pluripotency, differentiation and initiation of new tumors [13,14,15,16], in a process mediated by HIF-2α and its targets Oct-4, c-Myc and Nanog [17]. Targeting these progenitor cells should theoretically destroy a cancer, by preventing its further progression and that is one of the key aims of current therapeutic development [18]. To achieve this particular goal, it is essential to understand how cancer cells undergo EMT and become tumor-initiating cells resistant to radiotherapy or chemotherapy, and how they escape, invade and colonize other organs to form micrometastases. Multipotent tumor-initiating cells identified within many tumors, including melanoma and breast cancer, share important properties with their normal tissue stem cell counterparts with respect to self-renewal and differentiation capacity. The evidence is increasing that hematopoietic stem cells, embryonic cell lines and tumor-initiating cells are mobilized by hypoxia (and perhaps hypoglycemia) to undergo self-renewal and in many different cancers, hypoxia correlates with higher grades of more advanced malignancies with worse prognoses [16].

A major question for current cancer therapy is whether hypoxic-induced alterations in cancer cells will provide specific targets for selectively killing these cells, without affecting nearby normal cells. The recent evidence discussed below establishes a clear link between hypoxia and the mobilization of tumor-initiating cells as a critical driver of tumor progression from primary tumors to metastases. Often by the time cancers are detected, systemic micrometastases have become established, so that targets are needed that play essential roles in metastatic cells, but which are non-essential for normal cell growth and survival. Despite some improvements in cancer therapies, and surgical removal of primary tumors and draining lymph nodes, cancer recurrence and metastatic spread by residual disease remains a fundamental clinical problem, with many solid tumor types recurring at higher frequencies [19].

*Cancer eradication needs a set of targets with essential roles in metastasis that are important for not only inducing the pre-metastatic phenotype but are also essential for metastatic but not normal cell metabolism*.

## 2. Evidence for Amplified Mitochondrial Metabolism and Increased Oxphos in Metastatic Cancer

The Warburg hypothesis proposed that the driver of tumorigenesis is an insufficient cellular respiration (and ATP provision) caused by insult to mitochondria. The term “Warburg effect” is now used to describe that cancer cells and many cells growing in culture show higher levels of glucose fermentation (anaerobic glycolysis) and preferentially produce lactate even when sufficient O_2_ is present for cellular respiration (*i.e.*, the Pasteur Effect is absent in these cells). Warburg proposed this to be the root cause of cancer [20,21]. In further describing the origins of cancer, Warburg wrote: “*Probably chronic intermittent oxygen deficiency plays a greater role in the formation of cancer in the body than does the chronic administration of respiratory poisons. Any respiratory injury due to lack of energy, however, whether it is produced by oxygen deficiency or by respiratory poisons, must be cumulative, since it is irreversible*.” This prophetic example and the ensuing discussion about the consequences of injuring respiration eventually giving rise to metastatic tumors underlies the evidence presented here that the irreversible changes in mitochondrial function provide the information for developing selective drugs targeting these more lethal cancers.

In contrast to “healthy” cells which mainly generate energy from oxidative breakdown of several substrates such as pyruvate/lactate, glutamine/glutamate, free fatty acids, and ketone bodies [7,22] to form ATP by the metabolic process of oxidative phosphorylation (OxPhos), according to Warburg the driver of cancer cells should be interpreted as stemming from a lowering of respiration. Warburg proposed that a fundamental difference between normal and cancer cells was their glycolysis/respiration ratio—also known as the Warburg effect. This observation, based on studies of ascites tumor cells grown in high glucose, has pervaded the field ever since, although more evidence now indicates that, in spite of an enhanced glycolytic capacity, many cancer cell types exhibit functional mitochondria and that the principal site of ATP production in many cancers remains as OxPhos [23,24] even when cancer cells become hypoxic [25].

**Figure 1:**
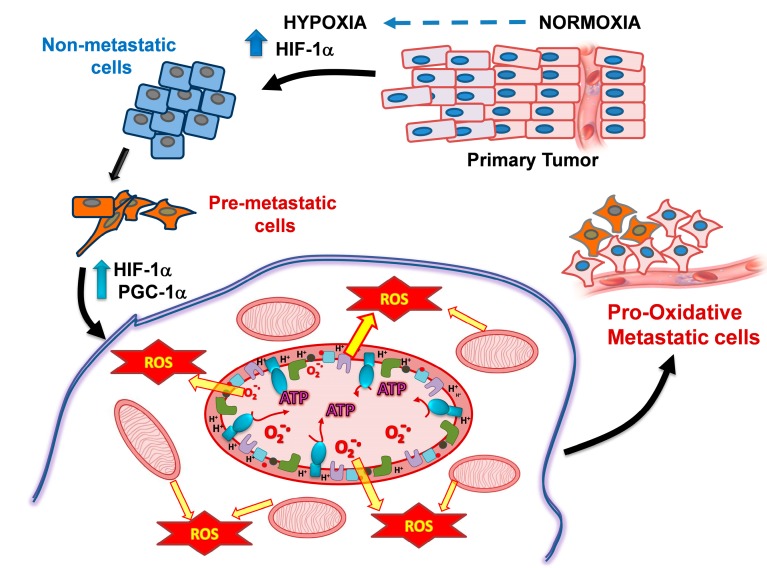
PGC-1α−mediated OxPhos activation and ROS generation promotes the metastatic cancer cell phenotype. Hypoxic non-metastatic cancer cells maintain a diminished level of mitochondrial metabolism. However, hypoxia causes PGC-1α (and HIF-1α, HIF-2α) overexpression which induces in pro-metastatic cells the generation of a higher number of mitochondria, increased OxPhos flux for ATP synthesis and increased ROS levels, all leading to the epithelial-mesenchymal transition and formation of highly metastatic cells. Abbreviations: OxPhos, oxidative phosphorylation; ROS, reactive oxygen species; HIF-1α, Hypoxia-Inducible Factor 1-alpha; PGC-1α, Peroxisome Proliferator-activated Receptor Gamma Co-activator 1-alpha.

Several recent reviews on targeting mitochondria-induced oxidative stress to treat cancers have been published [26,27,28,29,30,31]. However, our focus is on targeting metastasis due to its greater health implication and evidence that much higher levels of OxPhos and mitochondrial membrane potential (Δψ_m_,* i.e.*, the difference in electrical potential across the inner mitochondrial membrane) are hallmarks of the highly metastatic disease state (Figure 1).

First, we examine the evidence supporting a direct relationship between the coupling of increased Δψ_m_ with higher rates of OxPhos in metastastic cancer cells [32,33,34,35]. Thus, in 2005, Heerdt* et al.* clonally derived colon cancer cells that stably maintained higher Δψ_m _and also showed increased tumor invasiveness, VEGF and MMP-7 levels as well as chemoresistance [32]. Later, the same group examined colon and breast cancer cell lines derived from primary* versus* metastatic tumors to further establish that higher Δψ_m_ prevail in the more highly invasive, metastatic cells [33]. They proposed that the increased Δψ_m_ represented a marker for an acquired metastatic tumor phenotype.

*In vivo* studies by Bonuccelli* et al.* supported these earlier findings when upregulated OxPhos and TCA cycle activity were determined in human breast MDA-MB-231 cancer cells, promoted with 3-OH-butyrate and L-lactate and grown as xenografted metastases [34]. Later, they presented evidence of hyperactivation of OxPhos in human breast cancer biopsies by* in situ* enzyme staining for cytochrome c oxidase/Complex IV, Complex I and SDH/Complex II levels [35], although one should be aware that increased *protein content* (as revealed in this report by using activity staining assays with artificial redox dyes) may not be accompanied by increased *genuine* *enzyme activity and pathway flux*. A subsequent study focusing on metastatic tissue from human breast cancer lymph nodes showed amplified OxPhos function in the metastastic cells, whereas the surrounding stromal and immune cells were glycolytic, containing fewer mitochondria [36,37]. Hence, these studies refuted the conventional “Warburg effect” because it does not apply to the highly malignant metastatic phenotype [36].

### 2.1. PGC1-α Increases Metastatic Cancer Cell Metabolism

Further evidence for much greater mitochondrial capacity as a marker phenotype of highly metastastic cancer cells has been extensively reported, including the mechanisms causing such metastatic changes in cancer metabolism. Thus, studies of melanoma derived cell lines found subsets of cells with much higher Peroxisome Proliferator-activated Receptor Gamma Co-activator 1-alpha (PGC-1α or PPARγC1α ) protein levels and increased mitochondrial energy metabolism (increased CI-CIV respiratory chain protein contents) [38]. In addition, these cells exhibited greater survival under conditions of oxidative stress caused by ROS-inducing agents such as H_2_O_2_, PEITC, or piperlongumine, as well as a highly proliferative gene signature [38]. Although this study did not examine metastatic* versus* primary tissues, primary melanocytes were found to have low levels of PGC-1α expression, consistent with normal cells under normoxia.

A recent more thorough analysis of PGC-1α function, incorporating clinical analysis of human invasive breast cancers, found a strong correlation between PGC-1α expression, increased mitochondria, oxygen consumption, and OxPhos with the formation of distant metastases [39]. Moreover, silencing PGC-1α in cancer cells inhibited their invasiveness and metastatic potential yet had no effects on growth as primary tumors or the EMT program.

PGC-1α expression in cancer cells is induced by ROS (H_2_O_2_) and in turn works with the estrogen-related receptor α (ERRα) to induce expression of many genes involved in oxidative metabolism (including glycolysis, TCA cycle, OxPhos and lipid oxidation; Figure 1) and many of these overlap with those regulated by the hypoxic factors, HIFs, and their induced genes, such as VEGF. Also, PGC-1α activates TFAM (mitochondrial transcription factor A)-mediated mitochondrial biogenesis; MYC is another oncogene that also promotes mitochondrial biogenesis through TFAM. Analysis of PGC-1α function in normal cells showed that it also increases SIRT3 expression which in turn induces expression of ROS scavenging/detoxifying genes, including several components of the respiratory chain; glutathione peroxidase-1, superoxide dismutase 2, ATP synthase 5c, and cytochrome c [40,41]. To avoid over-interpretation of these last observations, it remains to establish whether such increased transcripts effectively translate into increased protein content and enzyme activities. Hence, PGC-1α appears to enable cells to overcome mitochondrial dysfunction in stressful conditions with higher Δψ_m_ and greater anti-oxidative capacity for coping with the increased levels of ROS production. Thus, the PGC-1α/ERRα signaling axis is highly relevant for many facets of cancer progression including invasion, angiogenesis and metastasis (reviewed in [42]).

## 3. Mechanism of Elevated ROS Production in Metastatic Cancer and the Role of Succinate

The extent of the electrochemical proton (H^+^) gradient or proton motive force (PMF, Δp) across the inner mitochondrial membrane (with Δψ_m_ as its main component) drives ATP production via Complex V, F_0_F_1_-ATPase or ATP synthase (reviewed in [43]). However, ROS production, as a by-product also indirectly depends upon the Δψ_m_, increasing when a build-up occurs in the levels of reduced intermediaries along the respiratory chain, such as FMNH_2_, FADH_2_, CoQH_2_, reduced iron-sulfur (Fe-S) clusters or cytochrome c Fe^2+^ by the respiratory complexes I-III. This situation occurs when the downstream functionality of the chain and particularly that of the ATP synthase becomes inhibited [43].

In contrast to the prevalent well-established view of *increased* ROS production in cancer cells induced by hypoxia during malignancy (reviewed in [7]), ROS produced by isolated mitochondria appears directly related to the pO_2_, *decreasing* under hypoxia [44]. These observations have led to suggestions that hypoxia-induced ROS production inside whole cells would then seem not related to respiratory chain activity but rather to depend on the availability of extramitochondrial factors [43] such as (a) decreased expression and activity of anti-oxidant enzymes (SOD, CAT, GPx, Prx III) [45,46,47]; (b) over-expressed NADPH oxidase [48]; or (c) increase in tumor-associated macrophages that produce ROS by secreting TNF-α, a protein that induces cellular oxidative stress under hypoxia [49]. However, the appropriateness of such conclusions drawn from studies with isolated mitochondria should be critically evaluated. This is because ROS production from mitochondria can be radically affected by the existing conditions such as the available level of O_2_ and critically depends on the substrate species, particularly the levels of succinate as a major driver of ROS production during hypoxia/ischemia [50,51,52,53].

Several studies have reported a direct link existing between increased Δψ_m_ and elevated ROS produced either by isolated mitochondria incubated with succinate [52,54] or when individual respiratory complexes are reconstituted into lipid vesicles [55]. The particular source of superoxide production that occurs along the respiratory chain depends on the type of oxidizable substrate that is predominately being used and the rate of electron transport [43,44,50,53]. Enhanced ROS production occurs when the electron carriers in the respiratory chain become stalled in the reduced state as a result of either the inhibition of OxPhos or respiratory chain complexes, or an excess of oxidizable substrate and saturation of the respiratory complexes. At this point, unpaired electrons residing in the ubisemiquinone bound to the Q binding sites of either of complexes I–III become available, promoting their reaction directly with O_2_ to produce superoxide anion as a toxic by-product of the O_2_ consumption by the respiratory chain (reviewed in [56]). Although such electron leakage from the respiratory chain can occur when the Δψm is low, ROS production is greatly reduced under such conditions and when ADP + Pi are readily available [42,50,51,52].

Studies with specific inhibitors of each of the complexes from I to IV, or with cells containing mutations affecting the activity of these complexes have shown that either of the complexes, when blocked, is capable of leaking electrons and producing significant levels of superoxide [57,58,59,60,61]. The levels of the mitochondrial ROS production are influenced by the redox state of the respiratory chain components, which are regulated by the transmembrane proton gradient (ΔpH) and Δψ_m_ [62,63]. Any agent that partially inhibits OxPhos, whilst substrates NADH for Complex I or succinate for Complex II are plentiful, will force the Δψ_m_ to rise and the electron transfer rate along the respiratory chain to diminish, promoting greater superoxide production via reaction of the electrons from ubisemiquinones or other redox components (e.g., FADH) in these complexes with O_2_.

In hyperpolarized cancer cell mitochondria, the electron transport chain stalls, possibly because of the lowering ADP and/or Pi levels due to the cytosolic metabolic demands of high glycolysis and conversion to ATP, inhibiting ATP synthase by limiting ADP and Pi availability in the mitochondria [56]. In addition, even under stressful conditions, internal ATP will continue to be pumped out of the mitochondria in exchange with external ADP by ANT [64]. Together with decreased passive proton permeability caused by increased cholesterol production and incorporation into both mitochondrial membranes [65], ATP synthase inhibition leads to a decreased consumption of the H^+^ gradient. However, the respiratory chain continues to oxidize substrates and pump protons across the inner membrane, resulting in an increased H^+^ gradient.

The importance of ubisemiquinones as the source of superoxide production was first recognized by Lehninger in studies of Complex III in heart mitochondria [66]. Alternative sites for ROS production from within the respiratory complex reaction centers, such as heme groups or prosthetic groups like FAD in Complex II [67] are also possible(reviewed in [43,68]). Despite debate over the actual sources of ROS, the fact remains that they are all capable of producing ROS under stressful, overloaded conditions and this point becomes very important for the anticancer therapies discussed in detail below.

## 4. The Role of Mitochondrial DNA (MtDNA) Mutation in Metastatic Cancers, Increasing ROS Production and Multidrug Resistance

It is widely recognized that many types of advanced cancers contain mutations or modifications either in their mitochondrially encoded DNA (mtDNA) or in the nuclear genes that encode mitochondrial proteins (reviewed in [69]). The cancer promoting effect of such mutations was supported when it was shown that inducing mitochondrial dysfunction by knocking down two subunits, GRIM-19 or NDUFS3 of Complex I increased ROS production, HIF-1α and EMT markers, promoting an invasive and more metastatic phenotype [70]. Although this study did not examine levels of OxPhos, it is consistent with the role of ROS in promoting greater malignancy [7]. Another study with a range of different tumor derived cell lines, using either* in vitro* or* in vivo* selection of superinvasive or supermetastatic tumor cells, showed that this selection was associated with increased OxPhos [70]. Noteworthy in the latter study was the increased levels of TCA cycle intermediates, succinate and 2-OG in the highly metastatic tumor cells [71]. Furthermore, inhibiting electron transport chain activity, induced with low levels of inhibitors such as rotenone, promoted mitochondrial ROS production and metastatic phenotypes* in vitro* and* in vivo*, which was prevented by treating the cells with the specific mitochondrial superoxide scavenger, mitoTEMPO or the mitochondrial ubiquinone antioxidant, mitoQ. Consistent with much of the evidence from other studies presented here, the authors [72] concluded that increased mitochondrial ROS was promoting the metastatic phenotype (reviewed in [7]).

Such studies are also consistent with earlier reports [73,74] where mutations induced in the mtDNA encoding subunit 6 of NADH dehydrogenase/Complex I caused increased ROS production and promoted cancer metastasis, which was inhibited by ROS scavenging agents. Another compelling study directly comparing gastric cancer cell lines differing only by their mitochondrial ROS production provided further solid evidence for ROS promoting invasive properties [75]. Adding to the role of mtDNA mutation, ROS and PGC-1α in cancer progression, Yao* et al.* showed that development of chemoresistance in the A549 human lung cancer cell line was associated with mutations in MT-ND2, encoding the mitochondrial Complex I subunit ND2 [76]. These mutations caused a 50% decrease in the NADH oxidoreductase activity of Complex I, which was compensated by increased mitochondrial biogenesis by PGC-1α, which was upregulated in cisplatin resistant cells [76]. Moreover, PGC-1α activation is coupled to increased HIF-1α stabilization by hypoxia [77]. In connection with these observations, it is relevant that hypoxia induced HIF-1α stabilization is also known to cause increased expression of the MDR-1 and MRP-1 multidrug resistance proteins in cancer cells [78,79,80] and that hypoxia-induced increases in A549/cisplatin drug resistant cells are reversed by RNA interference of HIF-1α expression [81] or by silencing PGC-1α [76]. Thus, PGC-1α and HIF-1α are tightly linked in hypoxia-induced malignancy.

The p53 tumor suppressor is another transcription factor induced by HIF-1α stabilization. Although experimental evidence could not be found suggesting a relationship between p53 and PGC-1α, soft tissue sarcomas (STS) with mutant p53 overexpress MDR-1 and maintain multi-drug resistance [82]. Reinsertion of wt p53 into cells harboring p53 mutations diminished MDR-1 expression, which in turn enhanced their anti-cancer drug sensitivity [82]. Interestingly, in null p53 tumors such as the cervical tumor derived HeLa cells or myeloleukemic HL60 line, no MDR-1 expression is observed [83,84], supporting the role for p53 in the regulation of MDR-1 expression. As a consequence, p53 null cells overexpress other multidrug-resistance systems such as the multidrug-resistance protein (MRP-1) [85].

## 5. Hypoxia and Changes in Mitochondrial Metabolism of Metastatic Cells as Targets for Their Preferential Killing

Hypoxia induced metabolic changes in cancer cells, including increased glycolysis and modifications to the TCA cycle and mitochondrial respiratory chain have all been extensively reviewed recently [86,87] and hence, are not discussed in detail here. The evidence for changes in mitochondrial function associated with metastatic cells was discussed in the previous sections. The following sections focus on the functions of drugs that selectively target the altered mitochondrial properties of metastatic cancer cells. Justification for developing drugs that are selective and major inducers of ROS production to activate cancer cell death has already been published [7,22,28,31,69,88,89,90,91].

Importantly, the recently reported role of succinate accumulation inducing elevated levels of ROS that cause damage during ischemia/reperfusion episodes in several organs/tissues [53] is also very relevant to the pathology of metastatic cancer cells selected by hypoxia. During ischemia, succinate accumulates to a greater extent than fumarate and malate due to favorable reversibility of the malate dehydrogenase, fumarase and succinate dehydrogenase reactions under hypoxia (and accumulation of NADH), which are further driven by purine nucleotide catabolism and the malate/aspartate shuttle. During reperfusion, accumulated succinate is oxidized by Complex II, inducing a toxic burst of ROS [53]. A similar sequence of events is likely to be involved when succinate accumulates in cancer cells during the development of the metastatic phenotype.

## 6. Advances in Mitocans with a Focus on Metastasis

The regulatory systems involved in mitochondrial ROS production in cancer cells were recently extensively reviewed in [29]. The focus here is on the actions of a selected set of drugs chosen either because they exhibit a range of exemplary properties or where the experimental evidence for their application to prevent or kill metastatic tumors is well established. Prime examples include the NSAIDs and the tocopheryl succinate family of MITOCANS (reviewed elsewhere [27]).

### 6.1. NSAIDs: Loading the Mitochondrial Gun in Highly Malignant Cells

**Figure 2:**
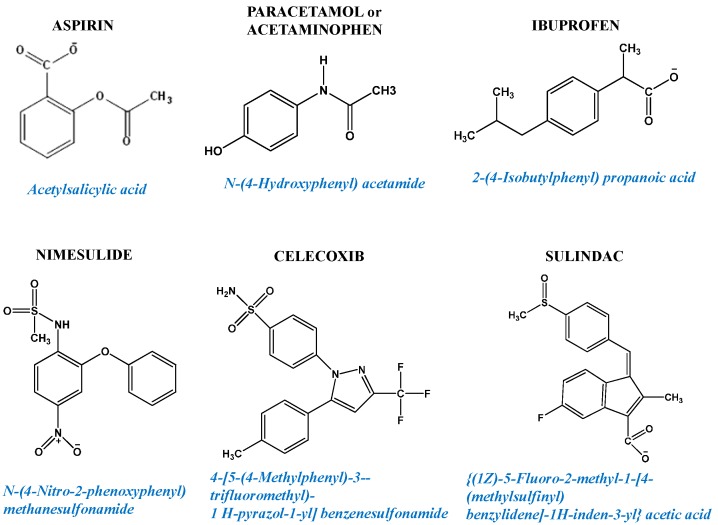
Examples of non-steroidal anti-inflammatory drugs (NSAIDs) chemical structures.

The commonly accepted medical use for NSAIDs (such as aspirin and paracetamol; Figure 2) is for the alleviation of pain and inflammation. They are generally characterized as inhibitors of cyclooxygenases (prostaglandin-endoperoxide synthases COX-1 and COX-2). These enzymes are normally activated by ROS and use arachidonic acid to produce the prostanoids, which then promote responses to pain and inflammation [92]. However, such a scenario grossly underestimates the potential of NSAIDs to treat metastatic cancers by lowering the guard of antioxidative defenses in the mitochondria. A word of caution though is that their clinical use as anticancer drugs must be tempered by their dose-related long-term side-effects causing damage to vital organs [93,94] and hence, high dosing should include mandatory monitoring of at least gastrointestinal, cardiovascular and liver functions [95,96,97].

Many longitudinal studies of patients taking low NSAIDs levels have consistently shown diminutions in cancer incidence in such populations by up to 30%–50% [98,99,100,101,102]. Consequently, the NSAIDs have been established in their cancer chemopreventative role. More importantly, many of the NSAID compounds have direct anticancer properties, working by destabilizing the redox systems in cancer cells to activate ROS-mediated killing, particularly of more advanced metastatic cancer cells. Some of this evidence was recently reviewed [103] and hence, the next sections predominantly consider the mitochondrial aspects of NSAID activity and their targeting of metastases.

#### 6.1.1. *In Vitro* Studies of NSAIDs as Anticancer Agents Inducing Apoptosis and Cancer Cell Death

Studies with celecoxib (Celebrex; Figure 2) showed potent antiproliferative effects and induction of apoptosis in a range of cancer cell lines [104,105,106]. These studies also showed that the mechanism for this anticancer action was independent of COX-2, or COX-1, the previously accepted drug targets. Further studies found that combining low levels (1–5 µM) of celecoxib with any of a range of standard anticancer drugs such as doxorubicin, vincristine, cisplatin, bleomycin or 5-fluorouracil increased the cytotoxic effects of the chemotherapy by up to ten fold, promoting apoptosis of human head and neck carcinoma cell lines* in vitro* [107]. Analysis of the effects of other NSAIDs on a range of human ovarian cancer cells subsequently showed that some were more potent at inducing apoptosis including flurbiprofen (4 nM), celecoxib (40 µM) and ibuprofen (200 µM) with combinations enhancing the cytolytic effects [108] and that celecoxib (10 µM) provided synergistic effects on paclitaxel (20 µM)-induced apoptosis of the human ovarian cancer cell line, OVCAR-3 [109]. It should be noted that the ability of NSAIDs to attenuate tumor cell growth and induce apoptosis does not correlate with tumor content of COX-1 or COX-2. It was suggested that other NSAID actions and mechanisms appear involved, particularly perturbation of the mitochondrial function (reviewed in [110]).

Comparisons of the relative anticancer activities and structure-function of the different NSAIDs have shown that their actions as anticancer agents usually involves use at higher drug concentrations than those required to inhibit the COX enzymes, with the anticancer effects mainly independent of COX inhibition (reviewed in [103,111]). The relative anticancer activities of different NSAIDs varied considerably with different types of cancer cell lines grown and analyzed* in vitro* [112,113,114,115,116] and the reasons for these differences in responsiveness are outlined in the next sections.

#### 6.1.2. *In Vivo* Studies of NSAIDs as Inhibitors of Xenografted Human Cancers

Several pre-clinical studies with human xenografted cancer cell lines treated with NSAIDs have now been reported. Aspirin (200 mg/kg/day) decreased RKO colon cancer cell growth and induced apoptosis [117]. Combining flurbiprofen (100 ppm; 100 mg/Kg food) and sulindac (200 ppm) added in the diet of xenografted mice was more effective than either drug alone at slowing growth of the SKOV-3 human ovarian carcinoma [108]. Diclofenac (18 mg/kg body weight) given intraperitoneally twice a week for 4 weeks or indomethacin (2.5 mg/kg) daily in their drinking water slowed the growth of HEY ovarian cancers [118]. Studies of human prostate cancer cell lines showed that sulindac (Figure 2) was effective at arresting growth of LNCaP in nude mice [119]. Combining celecoxib (5–10 mg/kg/day) with atorvastatin (5–10 mg/kg/day) was more effective at inhibiting prostate PC-3 or LNCaP tumors than either agent used alone [120,121]. The combination of atorvastatin *plus* celecoxib as daily i.p. injections significantly delayed the growth of LNCaP tumors in SCID mice and prevented androgen dependence from switching to independence by inhibiting IL-6 expression in the cancer cells [122]. Growth of orthotopic VCaP prostate tumors was strongly inhibited by treatment with the combination of atorvastatin and celecoxib [123]. Importantly, prostate cancer bone metastases were suppressed by oral celecoxib (15 ppm, equivalent to the standard human dose of 100 mg capsules). For colorectal cancer, indomethacin (50 mg × 2 daily) or celecoxib (100 mg × 2 daily) treatment for three days before surgery promoted significant lowering in the mRNA contents of several genes related with invasion and metastasis [124]. In summary, NSAIDs showed significant potential to enhance the anticancer actions of other chemotherapeutic drugs when used in combination and in a wide variety of cancers.

#### 6.1.3. NSAID Action on Nag-1 and p75(NTR) Affecting Cancer Cell Survival. Pre-Clinical Studies Where NSAIDs Were Used to Treat Xenografted Tumors

The NSAIDs have many effects on cancer cells beyond their ability to inhibit the COX enzymes. For example, many (sulindac, indomethacin, piroxicam) can competitively inhibit (in the micromolar range) dihydrofolate reductase (DHFR) activity, resulting in folate deficiency [125]. Indomethacin or NS-398 cause not only COX inhibition, but also a three- to six-fold decrease in expression of dihydropyrimidine dehydrogenase mRNA in tumor cells and xenografts of colon cancer cells [126,127,128]. Several other studies have reported on the effects of the NSAIDs on mitochondrial function using either isolated tissues or tumor derived mitochondria, or intact cancer or other non-cancerous cells [129,130,131,132,133,134,135,136]. Thus, diclofenac, mefenamic acid and piroxicam in the low to middle micromolar range behaved as mitochondrial uncouplers and inhibited OxPhos in rat kidney mitochondria utilizing either glutamate + malate or succinate, whereas dipyrone, acetylsalicylic acid and paracetamol required in the low millimolar range [129]. In extended studies, diclofenac and mefenamic acid at 2 µM, (concentrations well below the levels causing either uncoupling or inhibiting OxPhos) were 50-fold more potent than the classical uncoupler, salicylic acid at inducing Permeability Transition Pore Complex (PTPC) [136]. Similar results comparing a range of NSAIDs as uncouplers or inhibitors of OxPhos were obtained from studies using isolated rat liver or heart mitochondria or submitochondrial particles, intact hepatoma cells or isolated whole rat hearts [130]. In this study, nimesulide and meloxicam were more potent than diclofenac, indomethacin, piroxicam, naproxen or nabumetone for inhibiting OxPhos with glutamate + malate or succinate. Nabumetone inhibited respiratory Complex I but not Complex II activity and diclofenac inhibited ANT and ATP synthase activities, whereas naproxen did not affect mitochondrial function [130]. Thus, it can be summarized that the potency of the NSAID effects on mitochondrial function and killing of cancer cells does not correlate with their uncoupling activity or effects on OxPhos [130,136,137]. Neither did it relate to the severity of the NSAID effects on the gastrointestinal tract, suggesting that modifying mitochondrial function is not the direct cause of the high dose NSAID toxicity on normal cells [130].

On the basis of several other* in vitro* studies with a range of NSAIDs and different cancer cell lines, it was concluded that celecoxib and NO-aspirin in the 5–25 µM range were most effective in decreasing cell growth and inducing apoptosis at the lowest dosages and the ability of a particular NSAID to induce the expression of the p75(NTR) protein correlated with its anticancer cytotoxic activity [112,113,114]. The p75(NTR) protein is a member of the TNF related death receptor family, that contains a cytosolic death domain and acts as the common receptor for neurotrophins or proneurotrophins, is expressed by many tissues and correlates with pluripotency of stem cells, including cancer stem cells (reviewed in [138,139]. Several studies have correlated NSAID anticancer activity with their ability to induce cancer cell apoptosis after elevating expression of the p75(NTR) protein and induction of the NSAID activated (NAG-1) transcription factor encoding gene [140]. Interest in NAG-1 stems from its activation as a factor induced by many natural or synthetic pro-oxidative anticancer agents and that it is also induced by the early growth response factor (EGR-1) [141]. Expression of NAG-1 has been shown to increase lifespan by regulating energy metabolism, decreasing fat production by promoting thermogenesis, respiration, OxPhos and insulin/IGF-1/mTOR signaling [142,143]. Based on studies with colorectal cancer cell lines, it was proposed that the NSAIDs regulate EGR-1-mediated induction of ROS and NAG-1 to activate the intrinsic pathway of apoptosis [144]. However, in view of the following discussion this is unlikely to be the major death related mechanism involved in the anticancer activity of NSAIDs.

#### 6.1.4. NSAIDs in Clinical Trials of Human Cancer

Combination therapy in a Phase II study of carboplatin *plus* celecoxib in heavily pre-treated recurrent advanced stage ovarian cancer patients demonstrated that the treatment was well tolerated with promising response rates close to 30%, including 3 complete and 10 partial responses with median progression free survival and overall survival of 5 and 13 months, respectively [145]. Note that this study focused on advanced stage heavily pre-treated, recurrent cancers. In contrast, when used as a first-line therapy in a randomized Phase II trial of Stage IC to IV epithelial ovarian cancers, no significant difference between docetaxel and carboplatin* versus* docetaxel and carboplatin *plus* celexocib was found [146]. It is likely that this occurred because these cancers had not yet progressed to the point where they had become pro-oxidative with higher PGC-1α/HIF-1α levels, increased OxPhos and greater ROS production (see Figure 1). Hence, the NSAIDs may be better suited for use in targeting more advanced highly metastatic cancer cells after selection from prior rounds of chemotherapy, since the latter treatments engender higher pro-oxidative states than those found in early stages of cancers, before therapy.

## 7. Selective Anticancer Action of NSAIDs

### 7.1. The First Firing Mechanism–Destabilizing Essential Thioredoxin/Glutaredoxin Redox Systems

**Figure 3:**
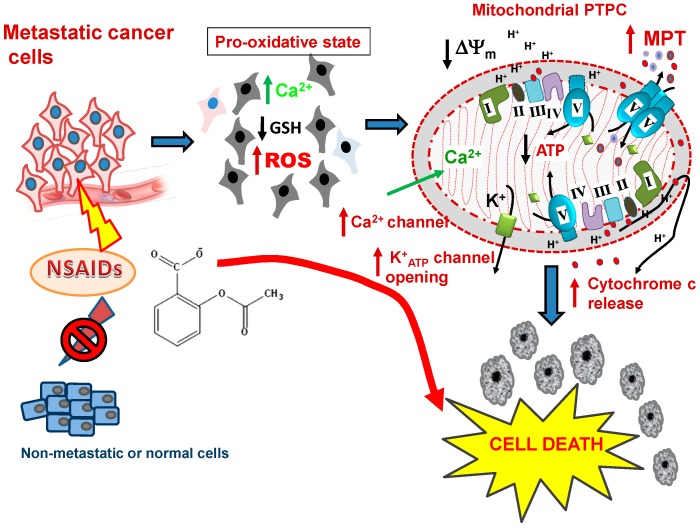
NSAIDs as mitochondrially targeted drugs in metastatic cells. After NSAID treatment, a heightened pro-oxidative status is induced in the metastatic cells, but not in non-metastatic tumor cells. The events ensuing in the metastastic cells include decreasing levels of GSH, critical changes in the Cysteine-thiol/disulfide bond status of key proteins and increasing ROS production, which is followed by Ca^2+^ overload, collapse of Δψ_m_, opening of the PTPC, release of cytochrome c and the other apoptosis-inducing factors leading to the death and elimination of these cells. Abbreviations: NSAIDS, non-steroidal anti-inflammatory drugs; GSH, reduced glutathione; ROS, reactive oxygen species, PTPC, mitochondrial permeability transition pore complex, MPT, mitochondrial membrane permeability transition.

The evidence is clear from many studies that one of the first intracellular events to occur in NSAID treated cancer cells involves metabolic changes (Figure 3), specifically in metastatic cells by decreasing levels of reduced glutathione (GSH) and increasing ROS production. This leads to an increased pro-oxidative state, and is followed by a decline in Δψ_m_, release of cytochrome c and finally, apoptosis [19,147,148,149]. The data also indicates that increased ROS itself also leads to increased EGR-1, followed by p75(NTR) and NAG-1 expression, providing a feed-forward amplifier circuit promoting cancer cell death. Moreover, structural modeling shows that sulindac or celecoxib can bind into the hydrophobic cleft formed by the BH3domain of the pro-survival protein, Bcl-xL [144]. This observation is consistent with reports that NSAIDs destabilize the pro-survival state by blocking and promoting degradation of Bcl-xL and Bcl-2 to release the pro-death BH3 domain only proteins, BID or BIM, thereby enhancing BAK or BAX activation, mitochondrial outer membrane pore (MOMP) formation and apoptosis of cancer cells [144,150,151,152]. The process of activation of MOMP has been reviewed recently [86] and it is one essential component of the death inducing mechanisms activated by the NSAIDs on cancer cells. The second component necessary for the full mitochondrial membrane permeability transition (MPT) is for the inner mitochondrial membrane channel or permeability transition pore complex (PTPC) to also become activated and that is discussed in detail below.

The earliest actions of NSAIDs in cells occurs by destabilizing the redox balance and antioxidant defense mechanisms of the thioredoxin and glutathione (GSH/GSSG ratio) systems, resulting in a greater pro-oxidative state with increased ROS production [153,154,155,156,157,158]. Several studies have shown that NSAIDs, such as aspirin and acetaminophen, in their reported therapeutic and cytotoxic concentration range, inhibit the enzymes involved in cellular redox control of both the thioredoxin [157] and glutathione systems, including glutathione reductase [156] and glutathione-S-transferase [158], resulting in increased GSSG and decreased GSH [153,154]. The net effect accentuates the pro-oxidative state and promotes the secondary increase in ROS production that enhances cancer cell death. In fact, the anticancer activity of a broad range of NSAIDs tested on human lung or leukemia cell lines correlates with their ability to be transported into cancer cells and inhibit glutathione-S-transferase [159].

The mitochondrial glutathione pool (~5 mM) is an essential redox system for protecting against oxidative damage, both by direct reaction with ROS or reactive nitrogen species (RNS) and as an electron donor for the antioxidant thioredoxin/glutathione/glutaredoxin/peroxiredoxin systems (reviewed in [160]). Hence, the glutathione redox system is crucial for cancer cell survival (reviewed in [161]).

### 7.2. Mitochondrial Redox Protein Modification by Glutathionylation

Glutathione S-transferase P (GSTP) is the GST superfamily member that is prevalently expressed in mammalian cancers, where it deprotonates glutathione to form a thioether bond with electrophilic substrates or promotes protein S-glutathionylation (reviewed in [162,163]). GSTP can use a wide variety of co-substrates and helps in detoxifying peroxidized lipids or facilitating breakdown of xenobiotics. Studies of isolated bovine heart mitochondria showed the reversible oxidation of reactive protein thiols by thiol-disulfide exchange, the extent of which was dependent on the GSH/GSSG ratio [164]. Thus, changes in protein thiol redox state, where the GSH/GSSG ratio drops during oxidative stress, is a redox signaling mechanism that regulates mitochondrial fate during induction of the MPT leading to apoptosis or necrosis of cancer cells.

The mitochondrial glutathione pool is distinct and separate from that of the cytosol [165], with mitochondria having their own glutathione reductase, glutathione peroxidases, and NADPH sources [166,167,168]. Studies of bovine heart mitochondria showed that the concentration of exposed protein thiols within the mitochondrial matrix is greater than that of GSH, suggesting that the interaction between the glutathione pool and protein thiols plays a critical role in mitochondrial antioxidant defense [160,164]. Up to 70% of the exposed protein thiols react with GSSG with some responding rapidly, within a minute or so, while the remainder undergoing redox transition gradually over 30 min. The more oxidized the glutathione pool (lower GSH/GSSG ratio), the greater the extent of protein glutathionylation.

Importantly, Complex I stood out as one of the very few mitochondrial membrane proteins to be persistently glutathionylated. Complex I has a role in mitochondria as a redox coupled proton pump, but it is also a source of ROS within cells [169], and it is highly susceptible to inactivating mutation during the oxidative progression of cancers (*c.f*. Section above). Although the redox based protein S-glutathionylation of Complex I subunits might participate in early signaling of the mitochondrial changes leading to apoptosis [164], rather the evidence indicates that this is a defensive mechanism diminishing oxidoreductase activity under conditions such as hypoxia where otherwise, Complex I would produce much greater levels of ROS. It has been shown that Complex I can produce superoxide while glutathionylated but was reversible because when the GSH/GSSG ratio was restored, superoxide production from Complex I returned to basal levels [170]. Many of the respiratory chain complexes and redox control systems are regulated by protein glutathionylation; the redox regulation of mitochondrial function by cysteine oxidation reactions has been recently extensively reviewed [171] and is outside the scope of this review.

## 8. The Pro-Oxidative Trigger Induced By NSAIDs

The potentially damaging actions of NSAIDs applied at high levels to normal cells can be used to provide greater insight into their cellular mechanisms of action. Thus, adding relatively high levels of acetaminophen (1 mM) to normal murine hepatocytes in cultures caused rapid (30–60 min) GSH depletion before oxidative stress and ROS production, leading to decreasing ΔΨ_m_, continuing to decline over ensuing hours in line with increasing cytotoxicity [172]. It was proposed that distinct phases occurred, first requiring acetaminophen metabolism as the GSH pools declined, perhaps due to direct drug interactions with GSH after conversion into the more highly thiol reactive metabolite, N-acetyl-p-benzoquinone imine (NAPQI) involving CyP action [172]. These compounds are also potent inhibitors of GST [173] which then led to the pro-oxidative phase of ROS production. A similar set of events was shown to occur in human HepG2 hepatocarcinoma cells, but in this case, with much lower doses (5–10 µM) of aspirin [153,154]. The anticancer effects of aspirin included decreased cellular glutathione (GSH) pool, increased ROS production, inhibited activities of Complex I, Complex IV and aconitase. Apoptosis was ultimately triggered by inhibition of OxPhos, decreased expression of the anti-apoptotic protein Bcl-2 and MPT activation [154]. The cytotoxic actions of aspirin were more marked when the HepG2 cells were first depleted of their pre-existing GSH levels by inhibiting glutathione synthesis with buthionine sulfoximine (BSO) [153]. Treating cells with N-acetylcysteine attenuated some of the apoptotic effects of BSO and aspirin, including collapse of ΔΨ_m_ and caspase activation, showing that the actions of aspirin were at least in part mediated by affecting GSH homeostasis.

Using additional agents to enhance the pro-oxidative state in cancer cells potentiates the anticancer cell activity of the NSAIDs. For example, pretreatment of human colon and lung cancer cells with sulindac (250 µM) enhanced killing by oxidizing agents such as tert-butyl hydroperoxide (TBHP) or H_2_O_2_ [174]. This effect did not involve COX inhibition, but resulted in a significant increase in ROS levels within the cancer cells and loss of ΔΨ_m_ leading to apoptosis. Aspirin or ibuprofen (either at 500 µM) was not as effective and the reason for this was not apparent. Acetaminophen treatment (1 or 10 µM) of the murine macrophage tumor derived J774 cell line caused decreased mitochondrial GSH pool, higher mitochondrial oxidative stress and ROS production and also acted to inhibit aconitase [175]. Thus, many of the NSAIDs are capable of activity at the micromolar levels to block the antioxidative defense systems in cancer cells.

## 9. Selectivity of NSAIDs for Preferentially Killing Malignant but not Normal Cells

Importantly, at therapeutic doses commonly given as anti-inflammatories, sulindac killed the human lung or colon cancer cells, but did not show effects on normal cells [174]. Similar results were shown with ibuprofen killing prostate cancer cells but not normal mouse fibroblast cells [113], emphasizing the distinctive nature of the NSAIDs for selectively targeting cancer cells. Although salicylate (10 mM) inhibited IL-lβ/TNF-α induced NF-kB activation in rat ventricular cardiomyocytes, it had no significant effects on the ROS levels in the control cardiomyocytes [176]. In a similar fashion, aspirin treatment of human K562 or Lucena erythroleukemic cell lines induced either their apoptosis or necrosis over the low millimolar levels whereas normal lymphocytes required 10 mM [177]. It has long been recognized that hepatotoxicity caused by exposure to high levels of NSAIDs, for example from gross overdosing [178], can be offset by administering N-acetylcysteine consistent with the drugs affecting oxidative stress mechanisms in a similar manner to their cytotoxic actions in cancer cells. Summarizing the results where comparisons have been made, killing normal cells requires much higher levels of NSAIDs than those sufficient for selectively killing malignant cancer cells. Given that metastatic cancer cells commonly express high ROS levels and pro-oxidative states, then the application of the NSAIDs to induce a further catastrophic ROS burst leading to cell death holds considerable promise [31,43].

## 10. The Firing Mechanisms for NSAID-Induced Cancer Cell Death: The Mitochondrial ROS Burst

**Figure 4:**
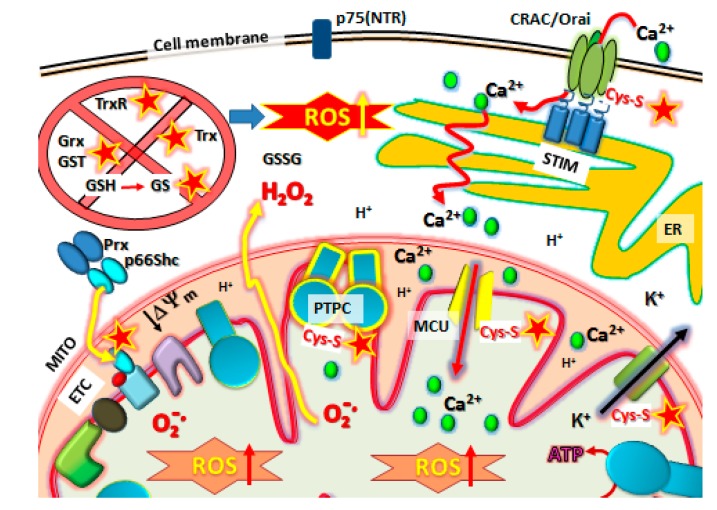
NSAID target sites for Cysteine-Thiol (Cys-S) reactivity in metastatic cells. NSAIDs have a multitude of potential targets, many mitochondrial. First, the antioxidative defense systems in metastatic cancer cells are knocked out by inactivating Grx, Trx, GST, TrxR, increasing the GSSG/GSH, before other targets are affected. These include reaction with Cys-S(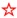) protein targets such as Orai1 to block SOCE, Prx/p66shc complexes to release p66shc which then translocates into the mitochondrial intramembraneous space binding to cytochrome C to promote ROS, modification of Kv ATP channels and mitochondrial Calcium (Ca^2+^) uniporter (MCU) regulators to cause mCa^2+^ matrix influx, increasing ROS production and promoting mPTP complex formation (PTPC) leading to cell death. Abbreviations: MITO, mitochondria. ER, endoplasmic reticulum. SOCE, Store-Operated Calcium Entry. STIM, Stromal Interaction Molecules.

It is clear that NSAIDs cause loss of redox balance by firstly lowering mitochondrial antioxidative defense systems, affecting mitochondrial function and producing a raised pro-oxidative state, which enables increased ROS production (Figure 3 and Figure 4). How then does elevated ROS bring about the death of metastatic cancer cells? There are likely to be several targets, which can act as triggers that fire the “loaded gun”—the capitulation and compromise of mitochondrial function precipitating the formation of the PTPC on the inner mitochondrial membrane as the initial irreversible step towards apoptosis or necrosis.

### 10.1. The p66Shc/PRX Couple

The p66Shc protein plays a dual role both as a trigger and mediator of ROS production and hence induces cell death. Thus, p66Shc is also a likely target in NSAID-treated cancer mitochondria that could trigger PTPC formation. p66Shc was identified recently as binding to cytosolic peroxiredoxin 1 (Prx1) [179]. However, p66Shc also becomes localized in the mitochondrial intermembrane space [180]. The 2-Cys Prx’s, including Prx1 and PrxIII, work as dimers and react with H_2_O_2_ which then converts them into disulphide-linked and inactivated dimers (or multimers) that are recycled into the activated dimers by two-stage reactions involving the thioredoxin (Trx) redox system. This includes NADPH, thioredoxin reductase (TrxR), and Trx itself, all three providing a key antioxidant system in the defense against oxidative stress through disulfide reductase activity to regulate the protein dithiol/disulfide balance in cells (reviewed in [181]).

The cytosolic and mitochondrial Trx systems along with the glutathione-glutaredoxin (Grx) system (NADPH, glutathione reductase, GSH, and Grx) both interact to control cellular redox homeostasis, the latter Grx system also providing a backup. The Trx system provides the electrons to the thiol-dependent peroxidases (Prx’s) to rapidly remove ROS and NO. The Prx’s are prone to hyperoxidation forming cysteine-sulfinic acids, inactivating them and requiring sulfiredoxin (SRX) to reduce them back to the thiol state. The TrxRs are less sensitive to oxidative stress than the Prx’s [182], although TrxRs have been recently shown to undergo electrophilic adduct formation that rapidly inactivates them via their reactive Cys-SH groups [183,184,185]. The Trx’s are very sensitive to oxidative stress when they are inactivated by oxidation induced by Prx and require reactivating by the Grx system [186].

Complex formation by p66Shc binding promotes dissociation of inactive Prx1 decamers to active dimers, thereby increasing Prx activity. At the same time, the Prx binding interaction decreases the ability of free p66Shc to enter into mitochondria to promote ROS mediated PPTC. Hence, p66Shc and Prx1 form a redox stress-sensing complex that keeps p66Shc in the cytosol at moderate oxidative levels. The p66Shc N-terminus forms a redox module responsible for apoptosis initiation, and undergoes activation through reversible tetramerization by forming two disulfide bonds upon oxidation (reviewed in [187,188,189]).

The relevance of NSAIDs to p66Shc activation is that the glutathione and Trx systems normally maintain p66Shc in the reduced and inactive state and when anti-oxidative systems are operating, p66Shc/Prx and GSH/Trx/TrxR interactions provide a thiol-based sensor system for regulating redox homeostasis. However, NSAID treatment deactivates the GSH/GST/Trx/TrxR anti-oxidative defense systems to initiate apoptosis by overloading these protective mechanisms with ROS. Consequently, knockout of p66Shc has been shown to prevent PTPC formation in neurodegenerative disease models [189] and p66Shc is one of the integral regulators of the mitochondrial oxidative stress that triggers ROS production leading to apoptosis [187].

Overexpression of p66Shc in the human colon cancer RKO cell line treated with H_2_O_2_ increased ROS production, whereas shRNA knockdown of p66Shc suppressed ROS. However, p66Shc induced ROS production also required the mitochondrial electron transport chain because rho zero cells (lacking mtDNA and a functional electron transport chain) did not show a p66Shc effect [188]. This is consistent with the demonstration that p66Shc in its serine-phosphorylated form sequesters electrons from the respiratory chain to generate ROS,* i.e.*, p66Shc interacts with and oxidizes cytochrome c-Fe^2+^ to form H_2_O_2_ [190] and, as a consequence, p66Shc collapses ΔΨ_m_ as a result of oxidative stress [180] (Figure 4). It is notable that the expression of p66Shc is required by p53 to increase ROS and induce cytochrome c release for activation of the mitochondrial apoptotic pathway [191]. The mitochondrial-targeted antioxidants, SkQ1 and SkQR1, also inhibited the p66Shc oxidative stress induced by H_2_O_2_, or serum deprivation. Taken together, these data indicate that p66Shc-dependent ROS production during oxidative stress involves the mitochondria to facilitate cancer cell death. Specifically, ROS activated p66Shc complexes form in the inner mitochondrial space, reacting with cytochrome c in the mitochondrial electron transport chain to promote further ROS production to trigger PTPC [187].

PrxIII located in the mitochondrial inner membrane space has been shown to act as an important ROS scavenger, protecting cancer cells from undergoing ROS mediated death and it is overexpressed in cervical [192] and prostate [193] cancers. It would be of interest to examine the relationship of p66Shc to PrxIII in this connection.

### 10.2. Kv-ATP Channels and Mitochondrial Membrane Depolarization

Another trigger firing the ROS burst to promote cancer cell death involves the mitochondrial K^+^_ATP_ channels (Kv’s for voltage-gated K^+^ channels). Kv1.3 and Kv1.5 channels have been well characterized, participating in the regulation of mitochondrial volume, ionic homeostasis, pH gradient and ΔΨ_m_. The mitochondrial ion channels and their function have been recently reviewed [194] as well as their identification as potential oncological drug targets to induce cancer cell death [195]. Treatment of A549 human lung cancer cells with dichloroacetate restored pyruvate flows into mitochondria and activated the Kv1.5 channel by an H_2_O_2_-dependent mechanism, leading to the PTPC opening and apoptosis [196] (Figure 4). Studies with specific inhibitors of Kv channels including clofazamine (which blocks Kv1.3 > Kv1.5) showed that these drugs induce Bax/Bak independent death of cancer cells and the ensuing apoptosis was proposed to be mediated via the mitochondrial Kv1.3 channel [197]. Oxidized LDLs with oxidized cholesterol increase Kv1.5 activity, decrease UCP2 (uncoupler protein 2) expression and rapidly activate mitochondrial ROS production in endothelial cells in a Kv1.5 dependent manner to promote extensive apoptosis [198]. Kv1.5 is also widely reported to be sensitive to changes in cellular redox balance [199,200,201,202].

The presence of mitoKv1.3 is critical for mitochondrial apoptotic events [203]. In particular, mitoKv1.3 was identified as a novel target of the proapoptotic protein Bax and a physical interaction between these two proteins was demonstrated in apoptotic cells [203,204,205]. Incubating isolated Kv1.3-positive mitochondria with Bax or the known Kv1.3 inhibitors MgTx, ShK or Psora-4 triggered typical apoptotic events including ΔΨ_m_ collapse, ROS production and cytochrome c release [203]. These effects were not observed in Kv1.3-deficient mitochondria. Mutation of the highly conserved Bax lysine 128, which faces the intermembrane space after mitochondrial insertion of Bax [206], abrogated Kv1.3 inhibition and the pro-apoptotic effects of Bax both in isolated mitochondria and intact cells expressing the mutant protein [204,205]. This data indicates that Bax binds to and inhibits Kv1.3 to trigger apoptosis.

Kv1.3 and Kv1.5 both have a Cys residue near the channel pore loop which is conserved throughout members of the family [207]. In addition, the alpha subunit Kv1 channel members are inhibited by binding with Kvβ1 subunits and the latter can be inactivated by oxidation of the seventh cysteine residue, likely due to formation of a disulfide bond, either between the inactivation gates or with other regions of the channel or Kvβ [208]. In addition, Kvβ is inhibited by oxidation of NADPH bound to the Kvβ1 channel [209].

Interestingly, pro-oxidants, such as tert-butylhydroperoxide (t-BHP, which oxidizes GSH and inhibits glutathione peroxidase [210]), caused GSSG release from rat liver mitochondria and induced formation of a low conductance pore or transient/reversible PTPC opening associated with release of Ca^2+^ and K^+^ but not H^+^ or altered ΔΨm [211]. Irreversible and complete PTPC opening with ΔΨm collapse required addition of exogenous micromolar Ca^2+^ or removal of Pi with the order of events in whole cells shown to be oxidative stress induced by t-BHP enhancing mitochondrial Ca^2+^ uptake, leading to increased matrix Ca^2+^, increased ROS formation, onset of the MPT, and cell death [211,212]. However, other pro-oxidants such as protein dithiol reactive cross-linking reagents like diamide [211], arsenic trioxide (AsO_3_) [213,214] or phenylarsine oxide [214,215] are highly active at inducing PTPC, depending on Bcl2 expression [216]. Adding oxidized glutathione (GSSG) during transient pore opening had no effect, whereas adding reduced glutathione (GSH) or reducing agents such as DTT strongly inhibited pore opening induced by t-BHP [214,215]. Since glutathione depletion occurs before PTPC activation by NSAIDs, these findings are particularly intriguing given that modulation of the fast-inactivating Kv channels can be achieved through oxidation-sensitive thiols. The use of t-BHP, is reversible in time or can be readily reduced by glutathione, and also involves the formation of Cys-Cys disulphide bonding [208,214,217,218]. It also raises the issue whether K^+^ channel mediated depolarization is linked to Ca^2+^ flows that activate the PTPC (Figure 4).

A direct interaction between NSAIDs and K^+^ channels has been reported for the large conductance K_ATP_ channels of the plasma membrane of aortic smooth muscle cells, with the following activation potency sequence: aspirin > flurbiprofen > indomethacin at micromolar doses (1–30 µM), and it promotes increased ROS and cellular hyperpolarization [219]. Thus, a direct effect of NSAIDs on mitochondrial K_ATP_ channels cannot be discarded and should be further explored.

## 11. The Final Stage: Activation of Metastatic Cancer Cell Death by PTPC Opening

PTPC when fully opened is a high-conductance channel located in the inner mitochondrial membrane providing the conduit for solutes of up to around 1500 Da to be released from the matrix (reviewed in [220,221]). The PTPC undergoes short-time openings producing transient depolarizations of Δψ_m_ [222] that can be induced by a range of physiological stimuli [223], while long-lasting openings cause permanent depolarization (Δψ_m_ collapse), loss of ionic homeostasis, depletion of matrix pyridine and adenine nucleotides, respiratory inhibition, and the generation of high ROS levels. In addition, matrix swelling occurs due to the high osmotic pressure, and the outer mitochondrial membrane disrupts with release of the repertoire of pro-apoptotic intermembraneous proteins, including cytochrome c. Thus, extensive PTPC opening is a point of no return, committing the cell to death, either by apoptosis (if enough ATP is present to sustain caspase activity) or via necrosis (when ATP is depleted) (reviewed in [86,221]).

At present, the precise composition of the PTPC is not clear, with two main viewpoints [224,225,226]. However, the evidence does seem clear that the PTPC is formed by components of the ATP synthase. An inhibitor protein, IF1, binds to the ATP synthase soluble catalytic core or F1 when the ΔpH and Δψ_m_ are low during oxidizable substrate deficiency or partial uncoupling by free fatty acids, and hence ATP hydrolysis is thermodynamically favored over ATP synthesis (reviewed in [227]). Thus, IF1 is important for inhibiting the ATP synthase hydrolytic activity during hypoxia or ischemia [228], whereas it has no effect on the ATP synthetic activity (at high Δψ_m_).

Cancer cells have been shown to escape ROS mediated death by inhibiting the ATP synthase hydrolytic activity via the overexpression of IF1 [229,230,231,232,233] and it has been proposed that after IF1-mediated inhibition of the ATP synthase, cancer cells survive by rewiring of energy metabolism and nuclear reprogramming due to the resulting increased ROS in their mitochondria [229,232,233]. The IF1-mediated ROS signal was superoxide based and IF1 was able to protect lung, breast and ovarian cancer cells from the apoptosis-induced action of staurosporine. The cellular H_2_O_2_ levels and the GSH/GSSG ratio were not altered by IF1 overexpression. However, quenching with the ROS scavenger MitoQ prevented protection against staurosporine-induced death. Interestingly, the intensity of the ROS-mediated response to IF1 overexpression directly correlated with the degree of protection against drug induced cell death. These findings suggest a role for IF1 induced mitochondrial ROS in promoting survival pathways in all cancer cells [229,232,233,234].

### NSAID Effects on Calcium (Ca^2+^) Channels, Role in MPT Activation and Increased ROS Production

NSAIDs can modulate Ca^2+^ uptake and release inside cells to inhibit cancer cell proliferation and although Ca^2+^ signaling plays an important role during cell proliferation, it is also essential for the activation of MPT leading to cell death [235]. The intracellular Ca^2+^ levels are regulated by two main types of ion channels, the voltage gated/voltage dependent Ca^2+^ channels (Ca_V_ or VOCE) with five known subtypes and the non-voltage Store Operated Ca^2+^ Entry (SOCE) channels (reviewed in [236]). The Ca^2+^ release activated Ca^2+^ channel (CRAC/Orai) subunit interacts with the Stromal Interaction Molecule (STIM) (see Figure 4) and the Transient Receptor Potential (TRP) channel to form the SOCE complex (reviewed in [237]). Many different types of TRPs exist that are relatively non-selectively permeable to cations, including sodium, calcium and magnesium and are predominantly found on the plasma membrane of mammalian cells.

The aspirin metabolite, salicylic acid in the 100 µM range was found to inhibit Ca^2+^ uptake into mitochondria by blocking SOCE, impairing the growth of human Jurkat T cell leukemia or HT29 colon cancer cells [238]. In studies of either rat aortic A10 as a model for vascular smooth muscle cells (VSMCs) or rat basophilic leukemia cells, salicylate and other NSAIDs, including ibuprofen, indomethacin and sulindac in the 100 µM range inhibited SOCE, but not VOCE induced mitochondrial Ca^2+ ^(mCa^2+^) uptake and inhibited the growth of the A10 cells [239]. It was proposed that the NSAIDs depolarized mitochondria, inhibiting SOCE by Ca^2+^ dependent inactivation of CRAC/Orai channels, thereby inhibiting cell proliferation. Other studies of VSMC ion channel regulation showed that 10 µM celecoxib, but not rofecoxib or diclofenac, activated the Kv7 channel and it inhibited the Ca_V_ L-type voltage-gated Ca^2+^ channel [240,241]. In a study of isolated rat kidney cell mitochondria respiring on NAD(P)H, paracetamol or ibuprofen did not affect mitochondrial Ca^2+^ (mCa^2+^) release even at 1 mM, whereas piroxicam or salicylate were poor inducers [135]. However, diclofenac or mefenamic acid in the 20–30 µM range were potent inducers of mCa^2+^ release [135] and the onset of the MPT associated mCa^2+^ release depended on the presence of 20 µM Ca^2+^ added exogenously and was sensitive to inhibition by Cyclosporin A (CsA).

In related studies providing further support, a critical role of free cytosolic Ca^2+^, but not uncoupling, was shown to be required for inducing MPT and cell death of immortalized human hepatocytes by diclofenac (500–1000 µM range) [242]. This involved forming oxidative metabolites of diclofenac by the action of the cytochrome P450 oxidase (CyP 2C9), most likely via production of benzoquinone imine intermediates [243,244]. Furthermore, a study of Ca^2+^ induced ROS production by isolated rodent brain and human liver mitochondria showed that high ROS production was linked to the triggering of MPT, independent of ΔΨm, NADH and respiration [245]. It was concluded that the Ca^2+^ induced MPT caused a dysregulated oxidative state with loss of GSH- and NADPH-dependent antioxidant ROS detoxification. Hence, Ca^2+^ channel release associated activation of the MPT and increased ROS production is important for inducing cell death, although the exact mechanism of Ca^2+^ interacting with, and its effects on the MPT has yet to be fully explained (Figure 4).

Two different models have been proposed to explain how Ca^2+^ regulates the PTPC based on studies proposing two different models of PTPC composition. In the first model, PTPC formation involves dimerization of the ATP synthase [226] with PTPC formation and opening in ATP-hydrolyzing mitochondria (constant ATP levels, low Δψ_m_) requiring twice the Ca^2+^ load to that of ATP-synthesizing mitochondria (constant ADP levels, high Δψ_m_). ATPase activity and knockdown of oligomycin sensitivity-conferring protein (OSCP) affected the Ca^2+^ sensitivity of PTPC formation and opening. Therefore, it was proposed that OSCP presence raises the level of Ca^2+^ required to open the pore [226]. These studies used the PTPC inducers Bz-423 and the peptidyl prolyl isomerase CyPD (which both act through OSCP at a region located on top of the lateral stalk in the matrix) to indirectly affect the permeability properties of the inner membrane. CyPD is the mitochondrial receptor for Cyclosporin A (CsA), which releases CyPD from its ATPase association. In relation to the enterocyte action of NSAIDs, using CsA on wild type mice or deleting CyPD in knock out mice were found to protect against gastrointestinal toxicity otherwise induced by ulcerogenic doses of diclofenac [246].

CyPD binding to the PTPC masks a possible PTP inhibitory site that otherwise binds to Pi which is proposed as a PTPC desensitizing factor, whereas mitochondrial matrix Ca^2+^ overload is a PTPC activator (reviewed in [220]). CyPD binds preferentially to the pore ATPase dimer, inhibiting ATPase activity and increasing its Ca^2+^ sensitivity (for PTPC activation), but CyPD is not a structural pore component and it binds to the OSCP subunit at the same site as the ATP synthase inhibitor benzodiazepine 423 (Bz-423). Bz-423 competes with CyPD binding to this site and both sensitize the PTP to Ca^2+^ and inhibit the ATPase activity, inducing apoptosis by promoting PTPC formation [226]. Decreasing OSCP expression by RNAi also made the PTP more sensitive to Ca^2+^ [226]. In the presence of Ca^2+^, addition of Bz-423 opened a channel showing electrophysiological equivalence of Ca^2+^-dependent currents that were indistinguishable from those of the PTPC and typical of the mitochondrial megachannel. Using either purified native dimers or monomers of the ATP synthase inserted into planar lipid bilayers and the inhibitors Bz-423, CyPD, oligomycin or resveratrol, it was shown that PTPC formation only occurred when dimers, not monomers were reconstituted which required Ca^2+^ and was affected by Pi and Mg^2+^ levels [226]. Channel opening was also inhibited by the ATP synthase inhibitor AMP-PNP (γ-imino ATP, a nonhydrolyzable ATP analog) and Mg^2+^/ADP.

The alternative PTPC model proposes that the c subunit, which forms a ring in the intramembraneous part of the Fo portion of the ATP synthase, may also form the PTPC since purified preparations of the c-subunit formed a voltage-sensitive channel when reconstituted in liposomes [224]. In this model, the F1 portion is released on activation of the PTPC. Prolonged high matrix Ca^2+^ overloads enlarged the c-subunit ring, disengaging it from the CyPD binding site of the ATP synthase F1, providing a mechanism for PTPC opening with oligomycin, which binds to the c-subunit [247] displacing CyPD and opening the pore. In contrast, recombinant F1 beta-subunit applied exogenously to the purified c-subunit in liposomes promoted pore closure, as did CsA binding. Knockdown depletion of the c-subunit using shRNA attenuated the Ca^2+^-induced inner membrane depolarization in cell mitochondria and inhibited Ca^2+^ and ROS-induced cell death, whereas increasing the expression, or single-channel conductance, of the c-subunit sensitized cells to death via persistent opening of the pore leading to rapid and uncontrolled mitochondrial depolarization [224].

Both models for the PTPC are consistent with the ATP synthase forming part of it [224,226,248,249] and chemotherapeutic agents can induce this enzyme to produce a high intensity ROS signal, ultimately triggering apoptotic cell death ([250,251,252], reviewed in [253,254]). The pro-survival protein Bcl-xl has been localized to the inner mitochondrial membrane, interacting with the ATP synthase to enhance its activity by decreasing futile ion cycling and proton leakage [228,255,256]. Thus Bcl-xl also regulates PTPC, linking events in the inner and outer mitochondrial membranes and knockdown of Bcl-xl showed that it was essential for the pro-survival effect of human IF1 inhibition of the ATP synthase [228].

## 12. How the NSAIDs Fire the PTPC. Targeting Critical Cys-Thiol Reactivity to Initiate the PTPC Opening and the Cell Death Cascade in Metastatic Cancer Cells

From all the information in the preceding sections, we can conclude that reagents such as NSAIDs lower the redox GSH/GSSG balance to produce a more highly pro-oxidative state in metastatic cancer cells. Many NSAIDs are converted by CyP enzymes to active analogues, such as quinones, quinone imines or other active derivatives that react with GSH or protein thiol groups. The reactive NSAIDs or their derivatives also modify Kv and Ca^2+^ ion channel gating leading to increased matrix mCa^2+^ that induces PTPC formation and consequent further enhanced ROS production upon opening of the PTP, firing a feed-forward loop that stabilizes the pore complexes into an open flowing conformation. The PTPC contains a reactive dithiol-disulphide exchange and studies with the thiol oxidant copper–*ortho*-phenanthroline and reducing agents N-ethylmaleimide and dithioithreitol (DTT) support pore opening by thiol reactive groups as a two-step event. The first event involves binding to the primary reactive -SH group(s) [257] causing a change in protein conformation and unmasking of a second oxidizable cysteine-thiol (Cys-SH) group in the next step enabling dithiol-disulfide exchange, increasing the probability of pore opening. This scheme is strongly supported by the finding that the opening of the PTPC is fully inhibited by reducing agents such as DTT or beta-mercaptoethanol (β-ME) after N-ethylmaleimide (NEM), proving that oxidation occurs downstream of the NEM reaction step in the pore-activating sequence (reviewed in [258])). The dithiol cross-linking agents arsenite (AsO) and phenylarsine oxide (PAO) (reviewed in [259] and see Section 10.2 above) have commonly been used to selectively interact with vicinal dithiols on proteins to form complexes structurally similar to disulfides and these agents caused a large shift in the apparent PTPC gating potential [260]. The shift was prevented by the monofunctional–SH interactive reagents NEM and monobromobimane, and reversed by DTT [260,261]. Similar effects occurred in de-energized mitochondria where AsO and PhAsO caused a drastic increase in the sensitivity of the pore to Ca^2+^ [214].

Hence, the specific dithiol(s) that control PTPC opening are a final key in the unleashing of the NSAIDs/ROS-induced apoptotic program that follows oxidation of mitochondrial GSH to allow targeting of highly pro-oxidative metastatic cells by triggering critical dithiol-disulfide exchange sites.

## 13. Pro-Drugs with Critical Cys-Thiol Reactivity—A Common Modality for Activating the PTPC in Metastatic Cancer Cells

The common theme that has emerged is that many of the NSAIDs such as diclofenac and acetaminophen are oxidatively metabolized by the cytochrome P450 CyP 2C9 to form reactive structures [262] that incapacitate the antioxidative (GSH/Grx/GST/Trx/TrxR) defense systems in metastatic cancer cells. The resulting pro-oxidative conditions will then allow the NSAIDs or their compound derivatives to react with critical Cys-thiol groups on ion channels or enzymes and modulate their activity causing mCa^2+^ release to promote PTPC formation (Figure 4). This Cys-thiol reactivity of NSAIDs is similar to that which has been found recently with a range of other anticancer drugs, including caffeic acid phenethyl ester (CAPE) and curcumin [263], the 4-substituted 4-hydroxycyclohexa-2,5-dienones (PMX464 “quinols”) [264] and triterpenoids such as 2-cyano-3,12-dioxooleana-1,9,-dien-28-oic acid methyl ester (CDDO-Me) [265,266], 2-methyoxystypandrone [267] and other benzoquinone like drugs with reactive carbonyls are all capable of electrophoretic addition to Cys-thiol groups.

By analogy, many thiol reactive inhibitors of NFkB activation do so by inhibiting the IkappaB kinase (IKK) beta (IKKβ) via reaction of the human enzyme Cys-179 thiol involving electrophoretic addition, with examples of similar reacting drugs including CDDO-Me [266], CAPE [268], PMX464 [266], 2-methoxystypandrome [267] and many others [269,270]. It is pertinent that the NSAIDs aspirin and salicylate have long been known to also inhibit NFkB signaling by blocking IKKβ activity [271], most probably via the same thiol-reactive quinone type of irreversible binding interaction. The anticancer drugs, CAPE and curcumin also form adducts with the Cys-thiol of Orai1 subunits of SOCE Ca^2+^ channels to inhibit them [263]. Numerous other examples exist in the literature of similar reactions, such as the metabolic activation of CAPE to reactive quinones to inhibit GST in melanoma cells [272]. It follows then that if, as known, the NSAIDs are metabolized into benzoquinone imines or other analogues capable of reacting with exposed Cys–thiols on ion channels (Figure 4) and with GSH to form inhibitors of GST or by directly binding the GSH/Trx antioxidant defenses, then these reactions will cause GSH levels to collapse and ROS levels to rise. This, together with the modified gating of Ca^2+^ and other ion channels causing them to open provides all of the essential componentry for the MPT (Figure 3 and Figure 4). It would also explain why blocking CyP 2C9 action in cells treated with diclofenac does not stop the membrane potential falling (uncoupling) but does stop the injurious effects of diclofenac [242]. Hence, active quinones, including ubiquinones [273,274] such as menadione (Vitamin K3) [275,276] will also act upon exposed Cys–SH groups, and on GSH reversing its antioxidant activity and promoting higher oxidative stress, Ca^2+^ release, ROS production and activation of mPTP ultimately causing metastatic cell death (Figure 4).

At this stage, we are probably only on the cusp of our knowledge of the total protein and enzymatic interactions and targets that the NSAIDs are capable of modifying in metastatic cancer cell mitochondria. Thus, the roles of the mitochondrial localized GSTs [277,278,279,280], voltage dependent anion channels (VDACs) on the mitochondrial outer membrane and adenine nucleotide transporters (ANTs) on the inner mitochondrial membrane [216,281,282,283] also may involve critical Cys-thiol residues and again could be affected by NSAID reactivity as many of these are involved in promoting the mPTP formation [284]. The NSAIDs can be added to the growing list of anticancer drugs that promote formation of or target the mPTP, an area recently reviewed [285] and which will not be further discussed here.

## 14. Conclusions

Drugs targeting mitochondria in metastatic cells to promote selective killing responses and prevent cancer progression are particularly significant in the fight to eliminate metastatic foci that escape surgery or are resistant to radiation or chemotherapy. We expect that additional advances will soon be made to further define mitochondrial alterations in order to inhibit cancer metastases and recurrence. In particular, the identification of selective triggers targeting critical thiols that activate the mPTP in metastasizing cells should make this goal achievable.

Although the NSAIDs can be toxic to normal cells such as liver and kidney, they show much greater selectivity and at lower concentrations target the highly metastatic cancer cells emerging from hypoxia and hypoglycemic stress with their heightened pro-oxidative states, as discussed throughout this review. The preclinical and clinical data provide sufficient evidence for their selectivity and specificity for inducing metastatic cancer cell death and should now pave the way for future therapeutic clinical trials to be undertaken with advanced stage human cancer patients using improved NSAIDs combined with other chemotherapeutic agents.

No doubt further research will continue to refine the basic mechanisms for hypoxic/oxidative changes in tumor cell mitochondria and provide novel targets to fight against cancer by understanding the specific nature of changes in their activity, some of which have been highlighted in this review. Despite improved understanding of cancer and some advances in treatments, high rates of metastases and tumor recurrence remain a worldwide problem and a considerable health burden on society. In Australia, the annual incidence of new cases of cancer is continually rising (now near 130,000 with a predicted increase to 150,000 by 2020; AIHW 2012) and cancer is a leading cause of death (49,000 in 2008 for Australia and New Zealand) with a huge social and economic impact (currently estimated in Australia as a $2 billion health cost). In Mexico and Central American Countries, 176,000 new cancer cases and 108,000 deaths were registered in 2008 [286], with breast cancer having the highest incidence in females [287]. Consequently, further research is needed to fully understand the effects of hypoxia and ROS activated changes during primary tumor progression that promote metastasis. The goal is to identify specific new and selective targets for anticancer therapy that will inhibit primary cancer, metastatic development and cancer recurrence.

## Abbreviations

CSC: cancer stem cell
EMT: epithelial-mesenchymal transition
MPT: mitochondrial membrane permeability transition forming the megachannel
NSAIDs: non-steroidal anti-inflammatory drugs
OxPhos: oxidative phosphorylation
PGC-1α: peroxisome proliferator-activated receptor Gamma Co-activator 1-alpha
PTPC: inner mitochondrial membrane channel or mitochondrial permeability transition pore (mPTP) complex
ROS: reactive oxygen species
TCA cycle: tricarboxylic acid or Krebs cycle
Δψ_m_: difference in electrical potential between the bulk water phases separated by the inner mitochondrial membrane
